# Hormonal, antioxidant, and body composition differences in national level male athletes: a comparative study of aerobic and anaerobic training

**DOI:** 10.3389/fspor.2025.1568873

**Published:** 2025-05-20

**Authors:** Manar Burhan Jaradat, Eyyad Maghayreh, Waqar Husain, Khaled Trabelsi, Adam Amawi, Haitham Jahrami, Hadeel Ali Ghazzawi

**Affiliations:** ^1^ Department of Nutrition and Food Technology, School of Agriculture, The University of Jordan, Amman, Jordan; ^2^ Department of Basic and Applied Sciences, Al-Balqa Applied University, Al-Balqa, Jordan; ^3^ Department of Humanities, COMSATS University Islamabad, Islamabad, Pakistan; ^4^ High Institute of Sport and Physical Education of Sfax, University of Sfax, Sfax, Tunisia; ^5^ Research Laboratory: Education, Motricity, Sport and Health, EM2S, LR19JS01, University of Sfax, Sfax, Tunisia; ^6^ Department of Movement Sciences and Sports Training, School of Sport Sciences, The University of Jordan, Amman, Jordan; ^7^ Government Hospitals, Manama, Bahrain; ^8^ Department of Psychiatry, College of Medicine and Health Sciences, Arabian Gulf University, Manama, Bahrain

**Keywords:** ghrelin, leptin, glutathione, glutathione peroxidase, aerobic sports, anaerobic sports

## Abstract

**Background:**

Competitive athletes exhibit distinct physiological adaptations depending on their sport type and training techniques. These variations influence body composition, hunger-regulating hormones, and antioxidant defense mechanisms, which collectively impact performance, recovery, and progression. This study aimed to compare body composition, serum levels of ghrelin, leptin, and glutathione (GSH), and their interrelationships in national level male athletes engaged in aerobic and anaerobic sports.

**Methods:**

Twenty competitive athletes (aged 17–38 years) were evenly divided into aerobic (AS) and anaerobic (AnS) sports groups. Body composition—including skeletal muscle mass (SMM), fat-free mass (FFM), fat mass (FM), and percent body fat (PBF)—was assessed using bioelectrical impedance analysis (BIA). Blood samples were collected after 8 h of fasting to measure ghrelin, leptin, and GSH levels. Dietary intake was evaluated using a 24-h recall. Group differences were analyzed using the Mann–Whitney *U*-test, and correlations were determined using Spearman's rank correlation, with statistical significance set at *p* < 0.05.

**Results:**

The AnS group displayed significantly higher SMM and FFM compared to the AS group (*p* < 0.05). Ghrelin levels were significantly lower in the AS group (*p* < 0.05), while leptin and GSH levels did not differ significantly between groups. Positive correlations were observed between ghrelin and both SMM and FFM (*r* = 0.585, *p* = 0.007), and between GSH and dietary protein intake (*r* = 0.476, *p* = 0.03).

**Conclusions:**

Anaerobic exercise enhances muscle mass, while aerobic exercise supports appetite suppression. Limitations include the small sample size and reliance on 24-h dietary recall. Future research should use larger, more diverse samples and explore combined training effects. Recommendations include incorporating resistance training into aerobic regimens to optimize muscle mass and monitoring protein intake to support antioxidant defense.

## Introduction

A structured exercise training program can modulate the efficacy of the appetite control system by compensating for increased hunger and improving the satiety response to a meal (1). Exercise induces hormonal changes as part of the body's response and adaptation to exercise-induced stress (2). These changes affect key hormones such as ghrelin and leptin (3). Leptin plays a multifaceted role by influencing body mass, reproductive function, proinflammatory immune responses, and angiogenesis (4). Regular exercise combined with low energy availability has been shown to reduce leptin levels (5). Athletes tend to achieve lower body weights to participate in weight-intensive sports or for fitness purposes (6), competitive and highly trained athletes who adopted diets of extreme caloric deficit for rapid weight loss had impaired performance (7).The relationship between long-term exercise and ghrelin remains inconsistent in the literature (8). Exercise types such as High-Intensity Interval Training (HIIT) significantly affect fat and post-exercise ghrelin levels, as reported in highly trained wrestlers when compared to a control group performing routine wrestling exercises (9). Regular exercise improves the body's antioxidant system (10). Aerobic, anaerobic, and combined exercise improves antioxidant activity with different response magnitudes (11). On the other hand, excessive exhaust training leads to ROS buildup, oxidative damage, and stress in chronically exhausted athletes (12), the exercise type, intensity, and training status of the individual contribute to oxidative stress (13). The antioxidant defense system is affected by ghrelin which increases the activity of key antioxidant enzymes such as superoxide dismutase (SOD) and catalase (CAT), which indirectly improves glutathione (GSH) levels (14). However, this study does not aim to study correlations between blood variables.

Competitive athletes exhibit distinct physiological adaptations in body composition, hormonal regulation, and antioxidant defense mechanisms based on their sport-specific training demands. While prior research has examined the individual effects of aerobic and anaerobic exercise on hormones like ghrelin and leptin, and on antioxidant status, a comprehensive investigation comparing these factors simultaneously in athletes from different sports disciplines is lacking. The primary aim of this study was to determine whether distinct hormonal and antioxidant profiles exist in competitive athletes competing at the national level, specializing in aerobic vs. anaerobic sports, and to assess how these profiles relate to body composition and dietary intake. We hypothesize that aerobic exercise can be correlated with lower levels of leptin, lower ghrelin levels, and a better antioxidant defense system. While this study provides valuable insights into hormonal and antioxidant profiles in elite athletes, the small sample size (*n* = 20) limits the generalizability of our findings. This study's findings will guide sports nutritionists to carefully plan diets for athletes, considering nutrients of concern based on exercise type, such as increasing dietary intake of antioxidants for improving antioxidant defense, increasing protein for preventing muscle loss, and focusing on rich fiber dietary sources to increase satiety. Coaches may imply adding combined exercises for muscle mass privileges from resistance and strength exercises, and hunger suppression related to aerobic exercises.

## Methods and materials

The study was conducted in Amman, Jordan. Participants were recruited conveniently based on their acceptance to participate in the study. A total of twenty male participants aged between 17 and 38 years were recruited, The aerobic sports (AS) group involved ten endurance sports including long-distance runners, and ten competitive athletes in the anaerobic sports (AnS) group included weightlifters and wrestlers.

The inclusion criteria involved engaging in long-term exercise for a minimum of 12 weeks. Adult males aged from 17 to 39 and adolescents were not included since they have fluctuated leptin levels (15), and they have variations in growth hormone (GH) levels GH is affected by the ghrelin hormone (16). Females were excluded because they have high alterations in appetite-related hormones, including ghrelin and leptin (17). Smokers were also excluded since smoking impacts ghrelin levels (18). Participants with chronic diseases were also excluded because the presence of chronic diseases is linked to suboptimal or insufficient glutathione levels, which are linked to oxidative-related disorders such as neurodegeneration, mitochondrial malfunction, and even cancer (19). Athletes were followed an unbalanced diet were excluded as intermittent fasting increases appetite, and high-fat diets are suggested to suppress hunger hormones (20, 21).

### Data collection and ethical approval

Approval for the study was obtained from the Institutional Review Board at the University of Jordan (IRB at UJ) with Decision Number 60–2022. The University of Jordan funded this study. The participants signed a consent form. The data collection process occurred at the AL-Hussein Youth City Club in Amman, Jordan, from September 22 to October 8, 2022, and included biochemical, anthropometric, and dietary assessments.

### Blood samples

Blood samples were collected from the participants in the morning between 9 and 11 am. All participants fasted for 8 h, and blood withdrawal occurred before training. Participants refrained from intense training in the 24 h. All blood samples were drawn at similar times of day and under comparable conditions to reduce variability in hormonal levels. A total volume of 3 milliliters was withdrawn and separated into two tubes for plasma and serum analysis. The samples were allowed to settle, centrifuged at 3,000°C for 20 min, and stored at −80°C until analysis at a laboratory in Amman.

### Anthropometric measurements and body composition

Weight and height measurements were recorded via electronic scales and nonstretchable tape. The participants wore minimal clothing, and their weights were measured to the nearest 0.1 kg. Accurate measurements were made to the nearest 0.1 cm while standing erect.

The skeletal muscle mass (SMM), fat-free mass (FFM), fat mass (FM), and percentage of body fat (PBF) were analyzed via the bioelectrical impedance analysis (BIA) technique, Inbody 270 was used. The standardized procedures for this test were taken into consideration. including standing up for 5 min before testing, avoiding eating before testing (participants were fasting), using the restroom before the examination, conducting the test before exercise, in the morning, and thoroughly cleansing the palms and soles with the InBody Tissue. During testing, making eye contact with the examinee was avoided. Athletes performed the test as soon they arrived at their training site between 9:00 and 10:00 am. The BIA technique showed high reliability in determining body composition (41). All tests were performed by the same investigator.

The study did not include objective measurements of training load for aerobic and anaerobic interventions. While exercise protocols were standardized by type and intensity, the absence of direct workload data prevents definitive conclusions about whether observed hormonal differences stem from exercise modality (aerobic vs. anaerobic) or unmeasured variations in mechanical or metabolic load.

### Dietary assessment

Dietary intake was assessed using a single 24-h recall, an interview-based 24-h dietary recall was conducted to ensure that the intake matched groups and reflected their usual intake. Dietary intake was assessed using a single 24-h recall, the previous day was a resting day. This method is subject to day-to-day dietary variability and may not fully represent habitual dietary intake, but we chose it due to its feasibility within the constraints of our study and its relatively low burden on participants, which facilitated recruitment. However, the 24-h recall is a reliable measure for assessing calorie and nutrient intake (22).

### Statistical analyses

This study used the Statistical Package for Social Sciences (version 22) for analysis. The independent samples Mann- Whitney *U*-test assessed differences in body composition and blood variables between groups. Spearman's correlation test was used to detect the correlations between variables, considering the significance level at a *p*-value < 0.05.

## Results

### Anthropometric measurements of body composition

Table 1 shows the body composition measurements expressed as ranks. The independent-samples Mann‒Whitney *U*-test detected the differences between groups, which compares the ranks, not the means. This test was used because the data was not normally distributed, and sample sizes were small, based on Shapiro–Wilk *p* < 0.05.

**Table 1:** Mean ranks and significant differences in body composition between athletes.

Variable	Mean rank Aerobic group *n* = 10	Mean rank Anaerobic group *n* = 10	Mann–Whitney *U* (significance) *P*-value
Skeletal muscle mass (kg)	6.70	14.30	0.003*
Fat-free mass (kg)	6.70	14.30	0.003*
Body fat mass (kg)	11.50	9.50	0.481
Percent body fat (PBF)	12.90	8.10	0.075

* The levels of skeletal muscle mass and fat-free mass significantly differed between the aerobic and anaerobic sports groups.

### Biochemical assessment

Table 2 shows the mean ranks of ghrelin, leptin, and GSH for groups, where the *p*-value expresses the significance of differences between groups, and an adjusted Bonferroni correction column to minimize type 1 error, and Cohen's d values to address the effect size of differences in variables between groups.

**Table 2:** Mean ranks and significant differences in blood levels of ghrelin, leptin, and glutathione between athletes.

Variable	Mean rank Aerobic sports *n* = 10	Mean rank Anaerobic sports *n* = 10	Mann–Whitney *U* Significance (*P*-value)	Bonferroni correction	Cohen's *d*
Ghrelin (pg/ml)	5.50	15.50	0.000*	0.000*	−6.21^a^
Leptin (pg/ml)	8.50	12.50	0.143	0.429	−0.73^b^
Total GSH µm	8.80	12.20	0.218	0.654	−0.24^c^

Data are presented as ranks. *n*: total number of participants.

*P*-values are estimated through Independent Samples Mann Whitney *U*-test.

^a^ *d* ≈ −6.21: This is an extremely large effect size, indicating a substantial difference between the aerobic and anaerobic groups in Ghrelin levels.

^b^ *d* ≈ −0.73: This is a medium to large effect size, suggesting a notable difference between the aerobic and anaerobic groups in Leptin levels.

^c^ *d* ≈ −0.24: This is a small effect size, indicating a minor difference between the aerobic and anaerobic groups regarding Total GSH levels.

* Indicates significant *p*-value.

The aerobic group had significantly lower levels of ghrelin compared to the anaerobic group. However, there were slight but insignificant differences in leptin and glutathione levels between the groups.

### Dietary assessment of all the participants

The 24-h recall dietary intake was analyzed via Elizabeth Stewart Hands and Associates (ESHA) Food Processor Nutrition Analysis Software (ESHA) and then statistically tested via the Mann‒Whitney test. There were no significant differences between groups in terms of caloric, protein, or carbohydrate contents, except for fat intake, which was significantly different.

### Detected correlations between variables

**Figure 1:**
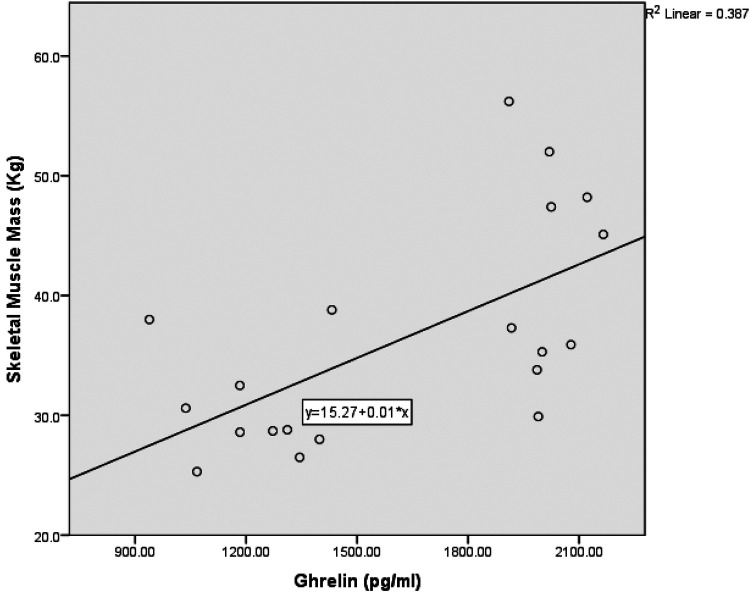
The correlation between ghrelin and skeletal muscle mass. (*N* = 20, *r* = 0.69, *p* = 0.001).

**Figure 2:**
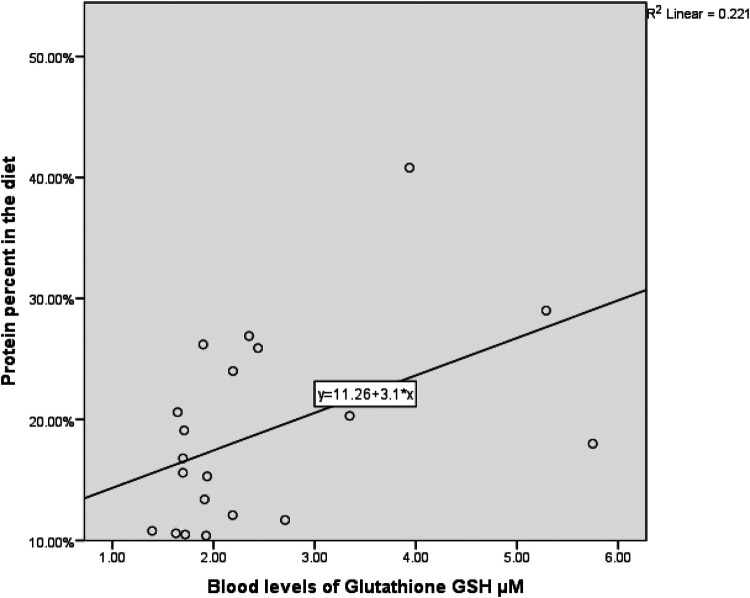
The correlation between blood levels of glutathione GSH µm and protein percent in the diet. (*N* = 20, *r* = 0.488, *p* = 0.034).

Due to the small sample size (*n* = 20), bootstrapped 90% confidence intervals were computed using the percentile method with 5,000 resamples to mitigate bias and account for non-normality. Spearman's rank correlation revealed a moderately significant positive correlation between skeletal muscle mass, fat-free mass (kg), and ghrelin (pg/ml) [*r* = 0.69, 90% CI (0.447, 0.820), *p* = 0.001], see Figure 1. This moderate positive correlation implies that higher ghrelin levels are correlated with higher muscle mass, supporting the preservation of muscle mass, especially in anaerobic sports athletes. Additionally, there was a significant moderate positive correlation between body mass index (BMI) and ghrelin (pg/ml) [*r* = 0.61, 90% CI (0.192, 0.836), *p* = .005]. A moderate positive correlation between skeletal muscle mass, fat-free mass (kg), and selenium dietary intake [*r* = 0.486, 90% CI (0.172, 0.830), *p* = .035]. This correlation suggests that selenium intake may indirectly impact muscle mass by enhancing antioxidant defense and muscle health. In addition, there was a moderate positive correlation between total glutathione (GSH) concentration and the percentage of protein in the diet [*r* = 0.488, 90% CI (−0.025, 0.796), *p* = 0.034], see Figure 2. This denotes the essentiality of dietary protein in the efficiency of the antioxidant system.

A strong negative correlation between age and ghrelin (pg/ml), [*r* = −0.83, 90% CI (−0.911, −0.647), *p* = 0.000], and a moderate negative correlation between training age and ghrelin (pg/ml) [*r* = −0.500, 90% CI (−0.764, −0.180), *p* = 0.029]. A significant negative moderate correlation between percent body fat (PBF) and protein intake (g/day) [*r* = −0.652, 90% CI (−0.861, −0.303), *p* = 0.002]. This indicates that higher protein intake improves body composition.

## Discussion

Body composition was distinct between groups. The anaerobic sports group had greater masses of skeletal muscle and fat-free tissue (kg), related to their exercise type, including strength (23) and resistance training (24) and due to the higher protein intakes (25). The lower muscle mass in aerobic exercise athletes (runners) aligns with the result of another study that compared body composition between strength and endurance athletes, as this type of exercise contributes more to cardiovascular endurance rather than increasing muscle mass (26). Fat mass was not significantly different between groups, as athletes tend to achieve lower body weights to participate in weight-intensive sports or for fitness purposes (6).

Exercise has a positive effect on ghrelin suppression (27). Our finding of significantly lower ghrelin levels in aerobic sports athletes aligns with the results of Plinta et al., who also reported that moderate aerobic activity reduces plasma ghrelin levels (28). This suggests that aerobic exercise may regulate appetite by suppressing ghrelin secretion (29). Higher energy requirements could explain the higher ghrelin levels in the Anaerobic Sports (AnS) group, as higher ghrelin levels were attributed to the higher demands for energy in resting situations (30). Ghrelin prevents muscle and protein breakdown (31). Animal studies examined this mechanism, but there was minimal research in humans. The correlation we found aligns with Nose et al., reporting that ghrelin administration mitigated the loss of skeletal muscle in patients with esophageal cancer after surgery (32). The mechanism between ghrelin and muscle breakdown prevention can be rationalized by the action of Acylated Ghrelin (AG) on the growth hormone secretagogue receptor (GHS-R), potentially affecting muscle health (33). Protein synthesis and minimized protein catabolism are affected by ghrelin through stimulating pathways such as AMPK and insulin signaling, as in mice. AMPK activation contributes to achieving energy balance to induce the anabolic process (34).

This study found a strong negative correlation between age and ghrelin levels, consistent with Nass et al., who reported an age-dependent decrease in circulating acyl-ghrelin levels (35).

This study addressed the lack of causality for the exact factor affecting ghrelin suppression, whether the type of exercise, lower muscle mass, or the older age of aerobic athletes. Although this study's findings were limited by a lack of in-depth exploration of these factors, it involved considering multiple variables and correlations, adding evidence to the literature that is minor in studies examining ghrelin and muscle mass. These findings are a field for further studies, and this leads to an alarming question: does low muscle mass lead to anorexia, sarcopenia, and food aversion?

Leptin levels were not significantly different between the groups, due to the significant differences in fat mass. The aerobic group had lower leptin levels, suggesting insufficient energy availability and low body fat (36). Prolonged exercise training causes an independent drop in leptin levels, regardless of adiposity alterations (37, 38). Furthermore, high-energy density meals can lower appetite and *ad libitum* energy intake in individuals participating in structured exercise training programs (1).

In this study, the distribution of Glutathione (GSH) was similar between the groups. However, the anaerobic sports group presented higher mean levels of GSH than did the aerobic sports group. Exercise decreases oxidative stress (13) and with increased levels of GSH (39). GSH availability is correlated with increased glutathione peroxidase (GPx) and antioxidant system activity (40). A study by Lu et al. revealed that physically active individuals have better antioxidant defense systems than sedentary individuals do (10).

Moreover, aerobic sports athletes have insignificantly lower protein intake, which suggests that lean protein enhances glutathione status (19). Consistent with our results, we found a positive correlation between GSH levels and protein intake as a percentage of the diet (*r* = .476, *p* = 0.03). There was no correlation between the GSH level and the total intake of calories, carbohydrates, or fat.

This study focused on various exercises with distinct energy systems, aerobic and anaerobic, including competitive male athletes involved in long-term exercise. Few studies have investigated the levels of hunger hormones and GSH in athletes. The findings of this study can help sports nutritionists create a dietary plan for athletes and instruct coaches and athletes in performing certain exercises based on their athletic goals. Small sample size is one of the limitations of this study due to limited access to competitive athletes in the region and the high cost of biochemical analyses, so we were constrained to a sample size of 20 participants. Nevertheless, this study does not reflect a cause-and-effect relationship; rather, it is intended to provide a description and identification of the levels of the blood variables in different types of exercise. Detailed training workload data (e.g., weekly hours, session RPE) were unavailable, preventing direct comparisons between aerobic and anaerobic groups. These limitations are explicitly acknowledged to guide cautious interpretation of the findings and underscore the need for future studies to address these methodological gaps.

## Conclusion

This study illustrates the favorable effects of exercise on appetite regulation and antioxidant defense in competitive male athletes. Aerobic exercise correlated with reduced ghrelin levels, indicating its potential for appetite control. Both types of exercises enhance antioxidant capacity, higher glutathione levels were detected in anaerobic exercises. The positive correlations observed between skeletal muscle mass and ghrelin, and between protein intake and glutathione, highlight the complex relationship between exercise, hormonal balance, and nutrition. Future research should expand on these findings with larger, more diverse cohorts. Ultimately, athletes can optimize their training by strategically integrating both aerobic and anaerobic exercises to achieve specific goals, such as managing caloric deficits, cutting weight, increasing muscle mass, and optimizing protein intake.

## Data Availability

The raw data supporting the conclusions of this article will be made available by the authors, without undue reservation.
